# Factors associated with initiation of medical advanced cardiac life support after out-of-hospital cardiac arrest

**DOI:** 10.1186/s13613-016-0115-y

**Published:** 2016-02-11

**Authors:** Jean-Christophe Orban, Didier Giolito, Jordan Tosi, Franck Le Duff, Nicolas Boissier, Christophe Mamino, Emmanuelle Molinatti, Thai Se Ung, Yassine Kabsy, Nicolas Fraimout, Julie Contenti, Jacques Levraut

**Affiliations:** Medical Surgical ICU, Pasteur 2 Hospital, Nice University Hospital, 30 Voie Romaine, 06001 Nice, France; Department of Emergency Medicine and SAMU-SMUR, Pasteur 2 Hospital, Nice University Hospital, 30 Voie Romaine, 06001 Nice, France; Department of Emergency Medicine, Bastia General Hospital, 20604 Bastia, France; Department of Emergency Medicine, Antibes General Hospital, 107 avenue de Nice, 06600 Antibes, France; Department of Emergency Medicine, Cannes General Hospital, 15 avenue des Broussailles, 06414 Cannes, France; Department of Emergency Medicine, Princess Grace General Hospital, 1 avenue Pasteur, 98012 Monaco, Monaco; Department of Emergency Medicine, Grasse General Hospital, 28 chemin de Clavary, 06130 Grasse, France

**Keywords:** Out-of-hospital cardiac arrest, Termination of resuscitation rule, Medical resuscitation

## Abstract

**Background:**

Termination of resuscitation rule permits to stop futile resuscitative efforts by paramedics. In a different setting, the decision to withhold resuscitation by emergency physician could be based on different factors. We aimed to identify the factors associated with the initiation of a medical ACLS in out-of-hospital cardiac arrest patients.

**Methods:**

We prospectively collected the characteristics of all out-of hospital cardiac arrest patients occurring in a French district between March 2010 and December 2013 and managed by the emergency medical system. We analyzed the factors associated with the initiation of medical ACLS.

**Results:**

Medical ACLS was initiated in 69 % of the 2690 patients included in the register. ACLS patients were younger (69 years [55–80] vs. 84 years [77–90]) and more frequently men. A higher percentage of witnessed cardiac arrest and BLS were observed. Duration of no-flow was shorter in the ACLS patients, whereas BLS duration was longer. A higher proportion of shockable rhythm and application of AED were found in this group. Mains factors associated with the initiation of medical ACLS were a suspected cardiac cause (1.73 [1.30–2.30]) and use of an automated external defibrillator (1.59 [1.18–2.16]), whereas factors associated with no medical ACLS were higher age (0.93 [0.92–0.94]), absence of BLS (0.62 [0.52–0.73]), asystole (0.31 [0.18–0.51]) and location in nursing home (0.23 [0.11–0.51]).

**Conclusions:**

The medical decision to not initiate ACLS in out-of-hospital cardiac arrest patients seems to rely on a complex combination of validated criteria used for termination of resuscitation and factors resulting from an intuitive perception of the outcome.

## Background

Cardiac arrest is one of the leading causes of death in Europe and North America accounting for more than 600,000 cases per year [1, 2]. Despite improvements in the process of care, survival is poor. Basic and advanced life supports represent the central links of the chain of survival. However, the utility to initiate or continue cardiopulmonary resuscitation (CPR) is questionable as most of out-of-hospital cardiac arrest patients die on the scene. Moreover, many patients are transported to emergency departments during CPR and are declared dead soon after, exposing emergency medical services and the public to the risk of high-speed transport. It is sometimes possible to discontinue out-of-hospital resuscitative efforts when they are considered futile [3, 4]. Several terminations of resuscitation (TOR) rules have been evaluated so far. They recommend stopping resuscitation efforts in cases of non-witnessed cardiac arrest, absence of bystander resuscitation, no delivery of shock and absence of return of spontaneous circulation. However, most of these studies come from North America, involving medical technicians or paramedics [5]. In one study, the decision of TOR was made by an emergency physician via an online medical control based on the emergency medical services [6]. Interestingly, the rates of TOR were significantly different across the bases involved in this study. This reflects the absence of criteria or guidelines to help physicians in this situation as TOR rules are only validated in their original setting [7].

In France, pre-hospital management of emergencies such as out-of-hospital cardiac arrest is a two-tiered system. Indeed, rescuers give first-line basic life support (BLS) and can apply automatic external defibrillator (AED). Next, advanced cardiac life support (ACLS) is delivered by physicians from the SAMU system (the emergency medical service regulation center) who are sent on the field after medical dispatching by a call center. Unlike the paramedics in the North American system, physicians in the field can take into account different parameters in their decision to withdraw resuscitation. Therefore, it seems that in addition to consensual TOR criteria, different factors influence the medical decision such as the patient’s age, medical condition, or duration of cardiac arrest [8].

The aim of our study was to evaluate the factors associated with the initiation of a medical advanced cardiac life support in out-of-hospital cardiac arrest patients.

## Methods

### Study setting

The “Registre Arrêt Cardiaque 06” (registry of cardiac arrest 06) covers the Alpes-Maritimes County (Nice and its surroundings in the south of France) with a population of 1.1 millions and a surface area of 4299 km^2^. Population densities are highly variable corresponding to urban, suburban and rural areas.

French emergency medical services consist of two-tiered system coordinated by the SAMU system. Firemen located in many proximal fire stations give BLS; they are authorized to use AED. Emergency medicine physicians stationed in bases located in hospitals and fire stations provide ACLS. In case of out-of-hospital cardiac arrest, the dispatching center sends the closest BLS provider and simultaneously an emergency medicine physician supervised by the SAMU system. As medical bases are more distant than local fire stations, firemen arrive often first on scene and immediately start BLS. The BLS and ACLS providers follow ILCOR guidelines during resuscitation [7].

### Data collection

All emergency physicians involved in the pre-hospital care system entered prospectively in an electronic database the report of each intervention for out-of-hospital cardiac arrest. Variables collected were: age, sex, location, duration of no-flow, presence of bystander, duration of low-flow before arrival of the physician defined as BLS, presumed cause of cardiac arrest, first recorded rhythm and use of automated external defibrillator. The admission to hospital after a sustained return of spontaneous circulation and outcome at discharge were obtained. To ensure the completeness and validity of the data, 4 physicians checked weekly the match between the medical dispatch software and the database of the study. In case of missing or evidently erroneous data, they contacted directly the physician in charge for updating or correcting the database.

### Patient population

For the purpose of this study, we included a population of out-of-hospital cardiac arrest patients aged >18 years and presenting the following criteria: no obvious signs of death (rigor mortis), presumed duration of no-flow <1 h and Abbreviated Injury Scale (AIS) <6 in case of trauma. The study was approved as a medical assessment registry without request for patient consent.

### Statistical analysis

Demographic characteristics of patients were summarized with basic statistical analyses including median and IQR for continuous variables and number and percentage for categorical variables. According to ACLS initiation, patients were dichotomized in “ACLS” (ACLS initiated by an emergency physician) and “no-ACLS” (absence of initiation of ACLS by an emergency physician). Mann–Whitney tests were used for univariate comparisons for continuous variables, while Chi-square tests were used for univariate comparisons of categorical variables. We used a stepwise logistic regression to estimate the odds ratios and 95 % CI to determine the association between the selected factors and initiation of ACLS. For model building, we introduced selected variables from univariate analysis with *p* < 0.2.

We considered *p* < 0.05 as statistically significant. All analyses were performed using XLSTAT version 2013.2.01 (Addinsoft, New York, NY).

## Results

**Fig. 1:**
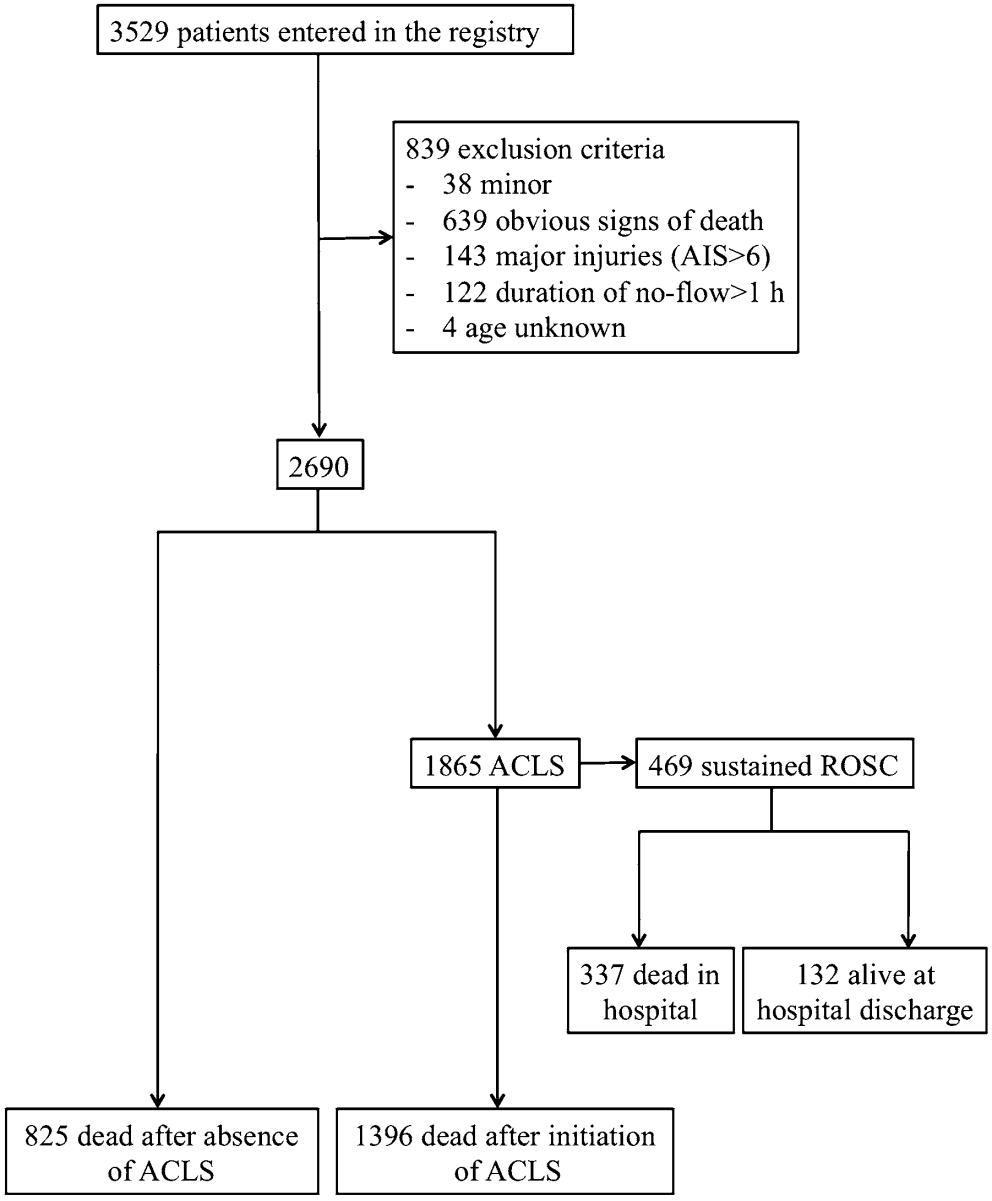
Flowchart of the study population

Between March 2010 and December 2013, 3529 out-of-hospital cardiac arrests were prospectively entered in the registry. After excluding patients <18 years, cardiac arrest with AIS = 6, obvious signs of death and no-flow time superior to 1 h, data of 2690 patients were analyzed (Fig. 1). In the study population, medical ACLS was started in 1865 patients whereas medical resuscitation was not attempted in 825 patients. ACLS patients were younger (69 years [IQR 55–80] vs. 84 years [IQR 77–90]; *p* < 0.01) and more frequently men (69 % [95 % CI 67–71] vs. 51 % [95 % CI 48–54]; *p* < 0.01). They presented a higher percentage of witnessed cardiac arrest (76 % [95 % CI 74–78] vs. 67 % [95 % CI 63–70]; *p* < 0.01) and BLS (78 % [95 % CI 76–80] vs. 62 % [95 % CI 59–65]; *p* < 0.01). The duration of no-flow was shorter (5 min [IQR 1–10] vs. 15 min [IQR 8–20]; *p* < 0.01), whereas BLS duration was longer (7 min [IQR 2–10] vs. 7 min [IQR 0–15], *p* = 0.04) in the ACLS patients. A higher proportion of shockable rhythm (9 % [95 % CI 8–11] vs. 0 % [95 % CI 0–1]; *p* < 0.01) and application of AED (16 % [95 % CI 14–17] vs. 2 % [95 % CI 1–3]; *p* < 0.01) were found in the ACLS group. The characteristics of the study population are reported in Table 1.

**Table 1:** Baseline characteristics of the study patients

	Study population	ACLS (*n* = 1865)	No-ACLS (*n* = 825)	*p* value
Age (years)	75 [60–84]	69 [55–80]	84 [77–90]	<0.01
Sex (% men)	64 [62–65]	69 [67–71]	51 [48–54]	<0.01
Witness (%)	73 [72–75]	76 [74–78]	67 [63–70]	<0.01
Basic life support (%)	73 [71–75]	78 [76–80]	62 [59–65]	<0.01
AED shock (%)	11 [10–13]	16 [14–17]	2 [1–3]	<0.01
Location (%)				<0.01
Home	69 [67–70]	66 [64–69]	81 [78–84]	
Nursing home	6 [5–6]	2 [1–3]	12 [10–14]	
Street	16 [14–17]	20 [18–22]	3 [2–4]	
Office	2 [1–2]	2 [1–3]	0 [0–0]	
Care facility	3 [2–3]	2 [1–3]	3 [2–4]	
Other	5 [4–6]	7 [6–8]	2 [1–3]	
First recorded rhythm (%)				<0.01
Spontaneous activity	5 [5–6]	7 [6–9]	1 [0–2]	
Asystole	88 [87–89]	83 [82–85]	99 [98–99]	
Shockable rhythm	7 [6–8]	9 [8–11]	0 [0–1]	
Suspected etiology (%)				<0.01
Respiratory	28 [26–29]	27 [25–29]	29 [26–32]	
Cardiac	32 [30–34]	38 [36–40]	18 [15–21]	
Trauma	5 [4–6]	7 [6–8]	1 [1–2]	
Miscellaneous	9 [8–10]	7 [6–8]	12 [10–15]	
Unknown	26 [25–28]	20 [19–22]	39 [36–43]	
No-flow (min)	10 [2–15]	5 [1–10]	15 [8–20]	<0.01
Low-flow (BLS) (min)	7 [0–12]	7 [2–10]	7 [0–15]	0.04

A logistic regression including the potential factors associated with the initiation of ACLS was performed. The absence of BLS, AED use, suspected cardiac cause, specific locations such as home and nursing home, and first recorded rhythm were the most significant factors associated with the initiation of medical ACLS (Table 2).

**Table 2:** Multiple logistic regression model with initiation of ACLS by a physician as the dependent variable

Variable	Odds ratio [95 % CI]	*p* value
Age	0.93 [0.92–0.94]	<0.01
Female sex	0.92 [0.82–1.04]	0.19
No-flow	0.95 [0.93–0.96]	<0.01
BLS duration	0.96 [0.94–0.97]	<0.01
Absence of BLS	0.62 [0.52–0.73]	<0.01
AED use	1.59 [1.18–2.16]	<0.01
Cardiac cause	1.73 [1.30–2.30]	<0.01
Respiratory cause	0.97 [0.74–1.27]	0.84
Trauma cause	1.43 [0.67–3.03]	0.35
Home	0.50 [0.25–1.00]	0.05
Street	1.79 [0.79–4.06]	0.17
Nursing home	0.23 [0.11–0.51]	<0.01
Asystole	0.31 [0.18–0.51]	<0.01
Shockable rhythm	2.13 [0.86–5.26]	0.10

## Discussion

In out-of-hospital cardiac arrest patients, we found several factors independently associated with the initiation of ACLS: age, suspected cause, location, duration of no-flow, initiation and duration of BLS, use of AED and first recorded rhythm. Thus, emergency physicians seem to initiate ACLS according to several criteria. They represent a combination of TOR rules validated in pre-hospital setting, and prognostic factors described in resuscitated patients from out-of-hospital cardiac arrest. The TOR rules have been reported in several studies concerning BLS as well as ACLS [3, 4, 9–11]. Both are considered conservative as only the presence of all criteria leads to stop resuscitation efforts. International guidelines do not recommend application of North American TOR rules out of their setting [7] even if they seem to perform well [9]. Thus, emergency physicians in the field are not supposed to take into account the complete list of criteria to decide starting ACLS. However, some parameters of the different ACLS TOR rules were associated individually with the decision, such as absence of witness, absence of BLS performed by a bystander and absence of defibrillation.

The other set of criteria corresponds to reported prognostic factors in out-of-hospital cardiac arrest patients [12–14]. First, the duration of no-flow was shorter in ACLS patients compared to no-ACLS patients. In the latter group, our study reports a median duration of no-flow of 15 min, exceeding the possibility of survival without intervention according to the model described by Larsen [12]. Recent studies showed similar results with a strong correlation between “downtime” and outcome [14, 15]. Although it seems that no-flow duration influences initiation of ACLS in our study, we emphasize that previous works correlated this parameter and outcome only in resuscitated cardiac arrest patients. So the validation of no-flow duration for the decision to initiate ACLS needs further research. Second, patients presenting cardiac arrest at home or in nursing home are less likely to be resuscitated compared to other places. The importance of location on cardiac arrest resuscitation and outcome has been emphasized. Cardiac arrest patients present a better outcome if it occurs in a public place [13, 16]. Actually, the circumstances explain this result with a higher percentage of shockable rhythm, the presence of bystanders and the application of AED [13]. Although poorly studied, the social pressure exerted by families or bystander could influence the medical decision in different ways. In a recent study, Morrison et al. reported a significant non-compliance with TOR rules explained mainly by the family distress [17]. However, families or relatives could influence the decision differently. Indeed, they represent an important source of information about the medical condition of the patient or do-not resuscitate order and hence can influence on the decision to resuscitate or not. At the same time, the decision to initiate resuscitation in the presence of relatives could influence their psychological outcome. Indeed, a high percentage of relatives of cardiac arrest patients present post-traumatic stress disorder [18]. A recent study demonstrated that their presence during CPR improved psychological variables [19]. Unfortunately, we did not collect the presence of families, relatives or caregivers, making it impossible to evaluate its impact on the intensity of care. Third, the initial rhythm correlates strongly with the initiation of ACLS in our study. Asystole was associated with the absence of ACLS, whereas a shockable rhythm or AED application was more frequent in the ACLS patients. This parameter represents also a described prognosis factor in out-of-hospital cardiac arrest in the field or after admission to intensive care unit [14, 20].

The key finding of our study is that ACLS and no-ACLS patients exhibit several significant differences. However, the results show a large overlap between the 2 groups, meaning that a large proportion of patients share similar characteristics. The key question is to understand why does a physician start ACLS or refrain to initiate resuscitative efforts in patients presenting similar characteristics. Apparently numerous prognosis factors (age, sex, no-flow, location, initial rhythm…) are incorporated to establish a prognosis in a few seconds. In the absence of recommendations, it sounds like a subjective decision based on the perception of the potential outcome of the patient. Thus, a decision to withhold ACLS in a patient having a potential good outcome would be a major ethical concern. Evidence-based medicine is not always translated strictly into clinical practice [21]. On the whole, our study illustrates a pragmatic application of evidence-based medicine: “research evidence, the clinical state and circumstances, as well as patient preferences and actions, all integrated together along with clinical expertise” [22].

While offering novel insights, some aspects of our study have to be interpreted with caution. The first limitation of our work is represented by the declarative nature of the study. Even if the emergency physicians filled out the form after each intervention, there is a possible discrepancy between what is declared and what is actually done. Second, because of the nature of the study, some parameters have not been collected. For example, it was impossible to evaluate several parameters such as the influence of the medical condition on care or the quality of BLS. Moreover, the influences of relatives or the social pressure during interventions have not been analyzed. These situations probably resulted in initiation of ACLS, whereas it was considered futile by the emergency physicians. Last, the organization of the French emergency medical service system is particular. In different countries, mainly paramedics provide BLS in the field, whereas in France, emergency physicians are always sent to the critical patient. Despite these shortcomings, we believe that our study raises important questions, which have to concern the medical community worldwide.

## Conclusions

The medical decision to not initiate ACLS in out-of-hospital cardiac arrest patients seems to rely on a complex combination of validated criteria used for termination of resuscitation and factors resulting from an intuitive perception of the outcome. Decision-making involves intimately the ethics, competence but also the presence of environmental factors that can perturb or even impair correct judgment. Further studies are needed to validate these criteria and evaluate their respective influence on the crucial decision to resuscitate out-of hospital cardiac arrest patients.
